# Relative Risk for Ehrlichiosis and Lyme Disease Where Vectors for Both Are Sympatric, Southeastern United States

**DOI:** 10.3201/eid2402.170962

**Published:** 2018-02

**Authors:** Marcia E. Herman-Giddens

**Affiliations:** University of North Carolina, Chapel Hill, North Carolina, USA

**Keywords:** tickborne disease, southeastern United States, ehrlichiosis, Lyme disease, public health, vector-borne infections, bacteria

**To the Editor:** The timely study on the relative risk for ehrlichiosis and Lyme disease in which the tick vectors, *Amblyomma americanum* and *Ixodes scapularis*, are sympatric notes that knowledge of tickborne diseases is “startlingly low” (*1*). The call for more research in diseases other than Lyme disease (LD) is long overdue. In the southeastern United States, 5 species of ticks bite humans (*2*). At least 11 associated human pathogens have been identified; all may cause tick paralysis (*2*,*3*).

This study also prompts comment on drawbacks. First, even where *A. americanum* ticks outnumber *I. scapularis* in high-incidence LD areas (*1*), there is no mentioned concern about inflated LD case numbers resulting from reporting patients with erythema migrans (EM) from *A. americanum* tick bites (*4*). Second, there is no evidence for or against a 1:1 transmissibility factor. Bites from infected ticks may not result in illness because of various factors. Subclinical cases may occur. Finally, LD may be reported more frequently because of EM occurrence compared with ehrlichiosis, which depends on laboratory criteria (*5*).

In addition, this study prompts pertinent observations. *A. americanum* ticks are known vectors of numerous pathogens and conditions, including several not yet reportable—for example, α gal allergy, Southern tick-associated rash illness, and Heartland virus—and no prevalence studies have been conducted, so their impact is unknown. It is notable that Monmouth County, New Jersey, USA, tests *I. scapularis* but not *A. americanum* ticks, which are more numerous, carry a greater number of pathogens, and are aggressive biters of humans.

Even though the Southeast United States has more tick species and tickborne pathogens, tick education campaigns, such as those conducted in the Northeast, are absent. The Southeast is experiencing human misery and economic impact from the increase in tick species and diseases. Attention to diseases other than LD is needed and is gratifying to see.

## References

[1] Egizi A, Fefferman NH, Jordan RA. Relative risk for ehrlichiosis and Lyme disease in an area where vectors for both are sympatric, New Jersey, USA. Emerg Infect Dis. 2017;23:939–45. 10.3201/eid2306.160528 28518034

[2] Stromdahl EY, Hickling GJ. Beyond Lyme: aetiology of tick-borne human diseases with emphasis on the south-eastern United States. Zoonoses Public Health. 2012;59(Suppl 2):48–64. 10.1111/j.1863-2378.2012.01475.x 22958250

[3] Herman-Giddens ME, Herman-Giddens DM. Retrospective case reports of two central North Carolina residents: Frequency of tick bites and associated illnesses, 2001–2014. N C Med J. 2017;78:156–63. 10.18043/ncm.78.3.156 28576950

[4] Wormser GP, Masters E, Nowakowski J, McKenna D, Holmgren D, Ma K, et al. Prospective clinical evaluation of patients from Missouri and New York with erythema migrans-like skin lesions. Clin Infect Dis. 2005;41:958–65. 10.1086/432935 16142659

[5] Centers for Disease Control and Prevention. Lyme disease case definition 2017 [cited 2017 Oct 4]. http://www.cdc.gov/nndss/conditions/lyme-disease/case-definition/2017/

